# Stressful life events in childhood and allergic sensitization* 

**DOI:** 10.5414/ALX01275E

**Published:** 2018-09-01

**Authors:** G. Herberth, S. Röder, A. Bockelbrink, T. Schäfer, M. Borte, O. Herbarth, U. Krämer, H. Behrendt, S. Sausenthaler, J. Heinrich, I. Lehmann

**Affiliations:** Environmental Immunology, Helmholtz Center for Environmental Research (UFZ), Leipzig, Germany

**Keywords:** children, stressful life events, neuropeptides, Th1/Th2 balance, allergic sensitization

## Abstract

Stressful life events evidently have an impact on development of allergic diseases, but the mechanism linking stress to pathological changes of immune system function is still not fully understood. The aim of our study was to investigate the relationship between stressful life events, neuropeptide and cytokine concentrations in children as well as the association between early stressful life events and atopic eczema (AE). Within the LISA plus (Life style – Immune system – Allergy) study, blood samples from children of 6 years of age were analyzed for concentration of the neuropeptides vasoactive intestinal peptide (VIP), somatostatin (SOM), substance P (SP) and the Th1/Th2 cytokines IFN-γ and IL-4. Life events such as severe disease or death of a family member, unemployment or divorce of the parents were assessed with a questionnaire filled in by the parents. Furthermore, lifetime prevalence of AE and incidence after the assessment period of life events were compared. Our data suggest that separation/ divorce of parents increase childrens risk of developing AE later in life. Children with separated/divorced parents showed high VIP levels and high concentrations of the Th2 cytokine IL-4 in their blood. Severe diseases and death of a family member were neither associated with neuropeptide levels nor with cytokine concentrations. Unemployment of the parents was associated with decreased IFN-γ concentrations in childrens blood but not with neuropeptide levels. Thus, the neuropeptide VIP might be a mediator between stressful life events and immune regulation contributing to the Th2-shifted immune response in children with separated/divorced parents.

German version published in Allergologie, Vol. 33, No. 2/2010, pp. 55-65

*Based on a lecture on the occasion of the 3rd German Allergy Congress, Erfurt, September 10 – 13, 2008.

## Introduction 

It is considered verified that psychological strain or stress influence the onset as well as the course of various diseases (e.g., asthma, allergies, autoimmune and tumor diseases) by modifying the capacity and regulation of the immune system [1, 2]. Dramatic life events such as death or severe disease of a family member as well as the separation of parents, but also other, relatively harmless, events like moving house are suspected to increase the allergy risk of affected children [3, 4, 5]. Apparently, the immune system mediates between stress on the one hand and allergic diseases on the other. Above all, allergic diseases like hay fever, allergic rhinitis, atopic eczema and asthma are based on the disequilibrium of the Th1/Th2 balance – with a reduced Th1 response and a strong Th2 reaction [6]. To date it is not completely understood how stress and psychosocial factors can influence the Th1/Th2 immune regulation. There are two ways of the nervous system to react to stressful situations: on the one hand the sympathetic nervous system releases catecholamines (epinephrine and norepinephrine), on the other hand the hypothalamic-pituitary-adrenocortical axis (HPA axis) releases glucocorticoids (cortisol) [7]. These neurohormones generally have a suppressing effect on the functioning of the immune system. According to recent scientific findings, neuropeptides produced by the sympathetic nervous system and by the HPA axis also interact with immune cells in a form of fine regulation of the immune system [8]. Among these neuropeptides are e.g. vasoactive intestinal peptide (VIP), somatostatin (SOM), substance P (SP), nerve growth factor (NGF) and neuropeptide Y (NPY) [9]. The nervous and the immune system influence each other via specific receptors. For example, T cells and dendritic cells have receptors for the neuropeptides VIP, SOM and SP. As a consequence it was demonstrated in in-vitro investigations that there is a direct interaction with T cells which might influence the Th1/Th2 balance [10, 11, 12]. Latest investigation shows that these neuropeptides are also released in stressful situations [13]. Therefore, our study aimed at investigating the relationship between stressful life events, neuropeptides (SOM, VIP, SP), Th1/Th2 regulation and allergic sensitization as well as the relationship between these life events and allergic disease (atopic eczema). 

## Methods 

### LISA study (Life style – Immune system – Allergy) 

The LISA study is a prospective cohort study comparing East and West Germany and aiming at investigating the impact of environmental and life style factors on the maturation of the infantile immune system and on the occurrence of atopic diseases. A total of 3,097 infants born in the period of 1997 – 1999 were recruited in the study centers in Munich (West Germany) (n = 1,467), Leipzig (East Germany) (n = 976) and Wesel/Bad Honnef (West Germany) (n = 654). 

In order to obtain a homogeneous study population, premature infants, children with postnatal diseases, congenital malformations or chronic diseases of the mother as well as children who were not of German origin were excluded. The parents had to fill in a questionnaire each year answering questions concerning parents social state (parents education), income, number of siblings, home furnishing, home moving, renovation activities, passive smoking, attendance at nurseries, pet keeping and occurrence of symptoms of atopic diseases. An additional questionnaire in the childrens 6th year of life (social questionnaire) covered information on stressful, critical life events. At the same time the blood levels of neuropeptides and cytokines were analyzed in the sub-cohort of children from Leipzig. The results presented here are mainly referring to these data and to the questionnaires filled in in the childrens 6th year of life. In addition, the relationship between atopic eczema in the childrens 4th year of life and the occurrence of previous dramatic life events was analyzed for the Munich and Leipzig cohorts. 

The parents participated in the study voluntarily. All parents of children involved in the study gave written informed consent. The study was approved by the ethics commissions of the Bavarian State Board of Physicians (Bayerische Landesärztekammer) and of the University of Leipzig. 

### Measurement of cytokines, neuropeptides and transcription factors 

Venous blood was taken with parents agreement after clinical examination of the children in the study center. The heparin blood for the determination of cytokines and neuropeptides was processed after a maximum of 4 hours storage at room temperature; the blood samples were stored at –80 °C until further analysis was carried out. The neuropeptides VIP, SOM and SP were determined using ELISA (Phoenix Europe, Karlsruhe, Germany) and the cytokines (IFN-γ, IL-4, IL-5 and IL-9) using cytometric bead array (CBA, BD Bioscience, Heidelberg, Germany) according to the manufacturers instructions and as described by Herberth and colleagues [14]. 

The transcription factors (GATA3, Tbet, FOXP3) and the regulatory factors (SOCS1, SOCS3) were determined after RNA extraction from 1 ml of heparinized blood (MagNA Pure LC System and MagNA Pure LC mRNA Isolation Kit, Roche Applied Science, Mannheim, Germany) using real-time PCR (Lightcycler, LC search Primer Kits, Roche Applied Science, Mannheim, Germany) as described by Herberth and colleagues [14]. 

### Confirmation of allergic sensitization 

Sensitization of the children was examined by detection of specific IgE (Immuno Cap System, Pharmacia Diagnostics). Total IgE and specific IgE against inhalable allergens (mixture SX1: allergens from Dermatophagoides pteronyssinus, cat and dog dander, pollen of timothy grass, rye, birch, mugwort and Cladosporium herbarum) as well as specific IgE against food allergens (mixture fx5: allergens from albumen (egg white), casein (cows milk), codfish, wheat flour, peanut and soybean) were determined. 

### Questionnaire investigation 

The details that were used for adjustment social economic status (calculated from the parents state of education), atopic family history, pet keeping (i.e., cat keeping), smoking during pregnancy, breastfeeding for a minimum of 4 months and season of birth originated from the questionnaire filled in when the child was born. 

In the childs 6th year of life the parents received a questionnaire covering the general life events (6-years questionnaire) and a further questionnaire concerning social environment/dramatic life events. Information on the number of siblings, noise exposure and home moving are derived from the 6-years questionnaire. The questionnaire concerning critical life events covered questions concerning death or severe disease of a family member, unemployment of parents and separation/divorce of parents. 

### Statistical evaluation 

Data were evaluated using Statistica7 for Windows (StatSoft Inc., Tulsa, Oklahoma, USA), StatXact 6.2 (Cytel Inc., Cambridge, MA, USA) and SPSS 13.0. For evaluation of differences between two metrically scaled, non-normally distributed groups Mann-Whitney-U-test was used. The neuropeptide concentrations were divided into quartiles (Q1 – Q4) and included into the model calculations. Cochran-Armitage test was used for trend calculation (relationship between neuropeptides and allergic sensitization). The Jonckheere-Terpstra test was used to calculate the trend of the relationship between neuropeptide concentrations and immune parameters (cytokines, relevant transcription factors). The risk to develop disease and allergic sensitization was calculated using adjusted logistic regression models, odds ratios (aOR) [4, 14]. Adjusted mean ratios (aMR) were calculated in order to demonstrate the relationship between stressful life events and neuropeptide and cytokine concentrations [15]. A Bonferroni correction of results was carried out. 

## Results 

### Characterization of the study population for calculation of the relationship between stressful life events in the first 2 years of life and atopic eczema 

For this evaluation the complete LISA cohort from the cities of Leipzig, Munich, Wesel, and Bad Honnef was taken into account (n = 2,420; 6th year of life). The relationship between stressful life events that took place in the first 2 years of life and the occurrence of atopic eczema in the 3rd and 4th years of life were determined. The most frequently reported dramatic life events in the first 2 years of life were severe diseases (17.5%) or death (8.4%) of a family member; separation/divorce of the parents (3.4%) or unemployment of one parent (2.7%) were less frequently reported. In total, the incidence of atopic eczema in the 3rd/4th year of life was 6.4%. 

### Relationship between stressful life events in the first 2 years of life and atopic eczema in the 3rd/4th year of life 

Separation/divorce was associated with a significantly increased (18.8% vs. 6.0%, p < 0.01), disease with a significantly reduced (2.2% vs. 7.2%, p < 0.01) and death with a trend to reduced (3.0% vs. 6.7%, p = n.s.) incidence of eczema in the 3rd/4th year of life. No relationship was found for unemployment (Table 1) [4]. 

### Characterization of the study population for calculation of the relationship between stressful life events and neuropeptide/ cytokine concentrations 

In Leipzig, 565 children still participated in the study in their 6th year of life. The parents of 324 children agreed to blood sampling and in 321 of these children Th1/Th2 cytokines, transcription factors and neuropeptide concentrations were measured in the blood. The parents of 479 children filled in the questionnaire covering social environment and stressful life events. For 234 children a completely filled in questionnaire and data concerning blood cytokine/neuropeptide concentration was available. Only these children were includwed in the calculations. In the 6th year of life the most frequently reported events in the Leipzig cohort were severe disease of a family member (9.8%), unemployment of parents (6.4%), separation/divorce of parents (2.6%) and more rarely death of a family member (2.1%). The other children had not experienced dramatic life events until their 6th year of life. 

### Relationship between stressful life events and neuropeptide/cytokine concentrations 

**Figure 1.**
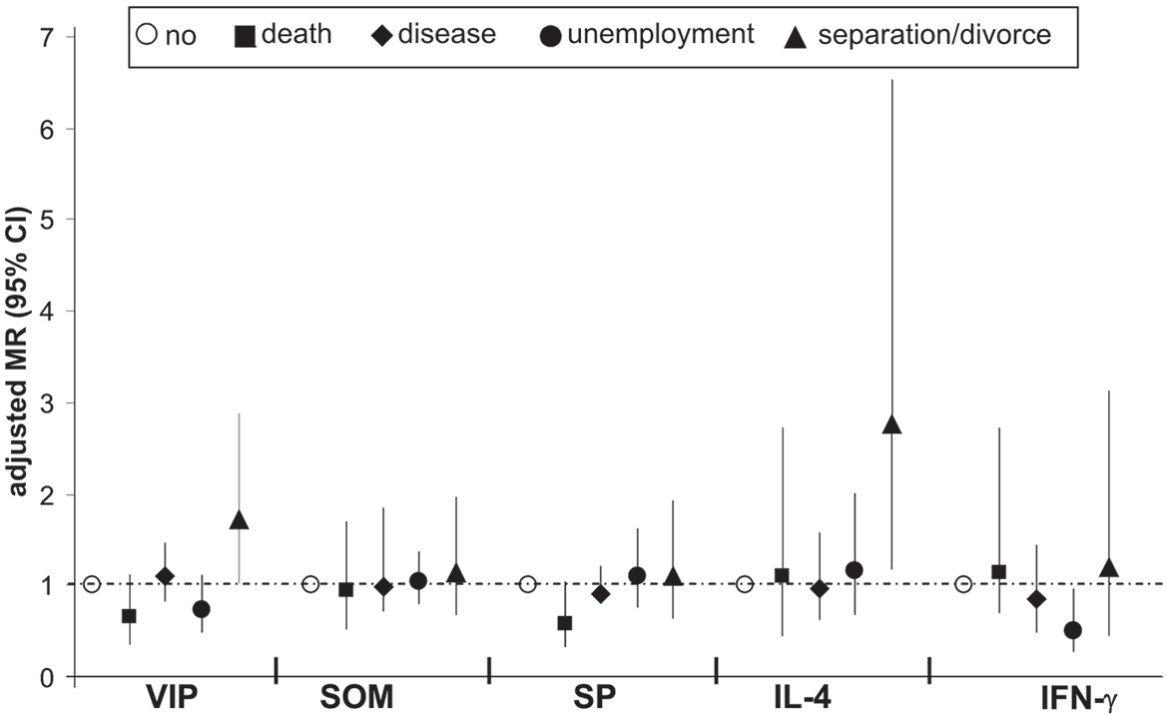
Relations hip between stressful life events in the 6th year of life and neuropeptide and cytokine concentrations in the blood of 6-year-old children. Data are shown as mean ratios (MR) and 95% confidence interval for VIP, SOM, SP, IL-4 and IFN-γ, ad jus ted for sex, atopic family history, mothers education and siblings.

In children whose parents reported separation/divorce in the year of blood sampling (6th year of life) significantly increased blood concentrations of the neuropeptide VIP (302 pg/ml) were measured as compared to children whose parents had not separated and who had not experienced other critical events (188.6 pg/ml) (Table 2). If separation/divorce of the parents had taken place longer than 1 year before, this difference was no longer detectable. Neither SOM nor SP was associated with separation/divorce of parents. Even after adjustment with the appropriate influencing variables (sex, atopic family history, mothers education, siblings) the relationship between VIP and separation/divorce of parents remained significant (adjusted mean ratio 1.72; 95% CI 1.02 – 2.88) (Figure 1). In the adjusted calculation model no further significantly relationships between stressful life events and neuropeptides were found. Children whose parents reported a home moving in the year of blood sampling had significantly increased blood levels of the neuropeptide VIP (data not shown, [16]). 

On the basis of raw data as well as in the adjusted model a relationship between separation/divorce of parents and IL-4 concentration in the blood of affected children was observed. In these children IL-4 concentrations were significantly increased (11.8 pg/ml) compared to children who had not experienced critical life events (5.7 pg/ml) (Table 2) (Figure 1). Furthermore, a relationship between unemployment of the parents and reduced IFN-γ concentrations in the blood of affected children was found (adjusted mean ratio 0.51; 95% CI 0.27 – 0.97) (Figure 1). As in the case of neuropeptides, these relationships were also only detectable, if the event had not taken place more than 1 year before blood analysis. 

### Relationship between neuropeptides, cytokines and allergic sensitization 

The relationship between the neuropeptides SOM, VIP, SP, and cytokines/transcription factors is presented in Tables 3, 4, and 5. A positive correlation between SOM and the Th2 factors GATA3 and IL-4 was detected. In contrast, there was an inverse relationship with the Th1 cytokine IFN-γ (Table 3). A negative relationship was observed between VIP and the Th1 transcription factor Tbet as well as between VIP and the regulatory factor SOCS3 (Table 4). 

The neuropeptide SP was positively associated with the Th1 cytokine IFN-γ and negatively associated with the Th2 transcription factor GATA3 and the regulatory molecule SOCS3 (Table 5). Of the 321 children whose blood was analyzed 92 (28.7%) reacted to inhalable allergens (SX1) and 41 (12.8%) to food allergens (fx5). The relationship between the neuropeptides SOM and VIP and allergic sensitization (specific IgE) is presented in Tables 6 and Table 7. The neuropeptide SP was associated with allergic sensitization. Taking into account potential influencing factors (adjusted model) it could be shown that SOM influences sensitization with food allergens. For children with high SOM values (4th quartile) the risk for sensitization against food allergens was almost 5 times increased (aOR 4.93, 95% CI 1.57 – 15.47), p trend = 0.001) (Table 6). Concerning single allergens an increased risk for sensitization against cows milk was observed when SOM values were increased (Table 6). Similar to SOM, sensitization against food allergens (fx5) and the single allergen cows milk was also observed for children with increased VIP values (aOR 3.5, 95% CI 1.25 – 9.82, p trend = 0.004 and aOR 4.0, 95% CI 1.02 – 15.67, p trend = 0.014 for cows milk). In addition, children with increased VIP concentrations had a 3 times increased risk to develop sensitization against rye (aOR 3.22, 95% CI 1.12 – 9.29) (Table 7). 

## Discussion 

Our investigation aimed at identifying factors that play an important role in the development of allergic sensitization and atopic diseases. We were interested in psychosocial stress factors as well as in mediators that might be of immunoregulatory relevance and that are released in stressful situations. 

Our investigation is the first to show a relationship between the blood concentration of neuropeptides (SOM, VIP and SP), immune regulation and allergic sensitization in children whithin an epidemiological study. It was described that in stressful situations there is an increased release of the analyzed neuropeptides [13, 17]. We could show that the expression of GATA3 transcription factor was higher in study participants with increased SOM values. GATA3 plays a crucial role in the differentiation of Th2 cells and in the development of allergies [18]. At the same time children with high blood SOM concentrations showed increased IL-4 and reduced INF-γ levels compared to children with low SOM concentrations. Similar to data from in-vitro studies [11] our results show that SOM is associated with a shift in the Th1/Th2 balance towards Th2. Simultaneously, a dose-dependent relationship between increased SOM concentrations and the risk of sensitization against food allergens and some inhalable allergens (timothy grass, rye) was detectable in children. According to in-vitro studies a Th1-inhibiting, anti-inflammatory effect can be attributed to the neuropeptide VIP [10]. In our study children with increased VIP values showed a reduced expression of the Th1 transcription factor Tbet. The risk for sensitization against a mixture of food allergens and also against single food allergens (rye) increased with elevated VIP concentrations. In contrast, no relationship between SP and a positive test against inhalable or food allergens could be shown. The Th1/Th2 balance – with high IFN-γ and low GATA3 values – was shifted towards Th1. These results also confirm the in-vitro data for SP: pro inflammatory reactions and secretion of Th1 cytokines [9]. 

The LISA study showed that neuropeptides are related to stressful life events and a shift in Th1/Th2 balance towards Th2. In the Leipzig cohort of the LISA study the VIP concentrations were increased in the blood of children whose parents reported separation/ divorce in the year of blood analysis. The blood levels of the Th2 cytokine IL-4 were also increased in these children [15]. These results support the LISA investigation on critical life events and the later occurrence of atopic eczema. It was shown that of all analyzed life events only separation/divorce of parents was associated to the incidence and prevalence of atopic eczema [4]. The neuropeptide VIP is released in stressful situations [13, 19, 20] and can cause a shift in Th1/Th2 balance towards Th2 [21]. As VIP is also related to allergic sensitization [14], our results suggest that VIP might be involved in the development of stress-related allergic diseases. In our study home moving was also associated with increased blood levels of VIP in affected children [16]. Contrary to this, dramatic life events like the death/severe disease of a family member or unemployment of the parents did not influence the neuropeptide concentration. Furthermore, Bockelbrink and colleagues [4] found out in the same study that severe disease of a family member or unemployment of the parents does not result in an increased risk of developing atopic eczema. According to this, children do not seem to perceive those kinds of life events as particularly stressful. 

Although our results have to be interpreted with caution due to the relatively low number of affected children, they do give valuable hints and a possible explanation for the mechanism that could be involved in the development of stress-induced allergic diseases. 

**Table 1.** Relationship between stressful life events in the first 2 years of life and atopic eczema.

	Occurrence of atopic eczema in the 3rd/4th year of life	
	OR (95% CI)	aOR (95% CI)
Life events		
Death of a family member	0.44 (0.16; 1.22)	0.44 (0.16; 1.21)
Severe disease of a family member	0.29 (0.13; 0.65)	0.30 (0.13; 0.70)
Unemployment	0.71 (0.17; 2.99)	0.76 (0.18; 3.19)
Separation or divorce	3.59 (1.69; 7.66)	3.78 (1.76; 8.10)

OR = odds ratio; aOR = adjusted odds ratio. AOR adjusted for sex, mother’s education, atopic family history, older siblings.

**Table 2.** Relationship between life events (within the 6^th^ year of life) and neuropeptide and cytokine concentration (raw data) in the blood of 6-year-old children (n = 234). Medians and 25^th^ – 75^th^ percentile.

	No event	Death of a family member		Severe disease of a family member		Unemployment of parents		Separation or divorce of parents	
[pg/ml]	n = 80	n = 5	p*	n = 23	p*	n = 15	p*	n = 6	p*
VIP	188.6 (141.7; 243.4)	125.2 (104.2; 125.4)	**0.044**	195.3 (125.4; 249.4)	0.874	175.2 (135; 244.2)	0.517	302 (206.5; 366.5)	**0.005**
SOM	31.8 (23.5; 44)	25.6 (17.9; 49)	0.837	24.2 (17.2; 55.7)	0.547	35.2 (24.8; 53.4)	0.690	29.6 (24.7; 35.3)	0.959
SP	217.8 (141.4; 260.6)	124.2 (107.4; 129.8)	**0.042**	166.1 (121.6; 261.9)	0.277	235.3 (111.2; 279.1)	0.951	184.6 (173.1; 228.2)	0.834
IL-4	5.7 (1.5; 9)	5.9 (5.8; 7.7)	0.741	5.4 (1.5; 8.9)	0.771	5.8 (1.5; 9.2)	0.831	11.8 (7.3; 25.6)	**0.023**
IFN-γ	8.9 (1.5; 13.9)	8.2 (7.2; 10.8)	0.841	7.1 (1.5; 12.1)	0.731	1.5** (1.5; 12.6)	0.126	5.8 (1.5; 14.7)	0.917

*p values from Mann-Whitney-U-test compared to the group without corresponding life events. Detection limit: 7 pg/ml for all neuropeptides and 3 pg/ml for all cytokines. **35% of values were under the detection limit.

**Table 3.** Relationship between SOM concentrations and T cell-regulating factors. Median for SOM quartile, 25^th^ – 75^th^ percentile and p trend value (p*). Significant values after Bonferroni correction are shown in bold font.

	1^st^ Quartile (3.50 – 21.50 pg/ml)	2^nd^ Quartile (21.50 – 30.30 pg/ml)	3^rd^ Quartile (30.30 – 42.80 pg/ml)	4^th^ Quartile (42.80 – 233.60 pg/ml)	p*
IL-4 mRNA	5.99 (2.52 – 18.47)	9.97 (5.18 – 34.97)	8.36 (4.79 – 18.76)	8.54 (4.55 – 20.85)	0.06
IL-4 protein	1.50 (1.50 – 7.40)	6.30 (1.50 – 9.20)	6.64 (1.50 – 11.8)	6.30 (1.50 – 9.40)	**0.001**
IL-5 protein	1.50 (1.50 – 2.08)	1.50 (1.50 – 2.36)	2.08 (1.50 – 2.50)	2.00 (1.50 – 2.34)	0.02
IL-9 protein	6.74 (2.00 – 14.28)	11.22 (2.00 – 17.2)	9.60 (2.00 – 20.14)	8.74(2.00 – 15.76)	0.100
IFN-γ protein	8.58 (2.00 – 15.18)	9.38 (2.00 – 13.66)	6.72 (2.00 – 14.41)	2.00 (2.00 – 12.86)	**0.001**
GATA3 mRNA	4.12 (2.36 – 9.98)	5.10 (3.33 – 9.90)	6.07 (3.83 – 10.58)	7.12 (4.07 – 16.44)	**0.0008**
Tbet mRNA	0.21 (0.06 – 0.46)	0.17 (0.04 – 0.38)	0.20 (0.10 – 0.35)	0.16 (0.08 – 0.29)	0.220
FOXP3 mRNA	0.51 (0.24 – 1.11)	0.58 (0.32 – 1.13)	0.52 (0.29 – 0.85)	0.37 (0.13 – 0.67)	0.007
SOCS1 mRNA	4.00 (2.13 – 13.37)	5.31 (2.72 – 17.75)	5.62 (3.49 – 8.97)	6.00 (3.90 – 17.28)	**0.003**
SOCS3 mRNA	2.77 (1.81 – 4.78)	3.37 (2.15 – 5.67)	3.94 (3.04 – 6.13)	2.97 (0.81 – 4.06)	0.224

**Table 4.** Relationship between VIP concentrations and T cell-regulating factors. Median for VIP quartile, 25^th^ – 75^th^ percentile and p trend value (p*). Significant values after Bonferroni correction are shown in bold font.

	1^st^ quartile (3.50 – 141.20 pg/ml)	2^nd^ quartile (141.20 – 196.7 pg/ml)	3^rd^ quartile 197.7 – 249.2 pg/ml)	4^th^ quartile (249.2 – 561.2 pg/ml)	p*
IL-4 mRNA	8.33 (3.19 – 21.54)	7.30 (3.91 – 15.25)	9.40 (4.11 – 23.4)	9.57 (5.63 – 27.13)	0.06
IL-4 protein	5.94 (1.50 – 10.08)	4.80 (1.50 – 9.56)	5.85 (1.50 – 8.45)	5.76 (1.50 – 9.40)	0.374
IL-5 protein	1.50 (1.50 – 2.36)	1.50 (1.50 – 2.34)	1.12 (1.50 – 2.36)	1.50 (1.50 – 2.34)	0.463
IL-9 protein	9.60 (2.00 – 17.22)	9.90 (2.00 – 15.76)	9.17 (2.00 – 17.22)	7.80 (2.00 – 15.76)	0.493
IFN-γ protein	9.55 (2.00 – 15.68)	5.68 (2.00 – 10.77)	7.31 (2.00 – 13.07)	8.54 (2.00 – 13.88)	0.353
GATA3 mRNA	4.69 (3.36 – 9.58)	6.55 (3.09 – 13.32)	6.82 (3.70 – 12.42)	5.69 (3.30 – 12.05)	0.09
Tbet mRNA	0.22 (0.10 – 0.44)	0.21 (0.12 – 0.34)	0.12 (0 – 0.29)	0.16 (0.07 – 0.30)	0.03
FOXP3 mRNA	0.53 (0.25 – 1.07)	0.52 (0.30 – 1.04)	0.47 (0.13 – 0.83)	0.43 (0.16 – 0.83)	0.06
SOCS1 mRNA	5.00 (3.38 – 14.85)	4.89 (3.28 – 9.50)	5.59 (3.31 – 13.37)	5.96 (2.97 – 18.92)	0.155
SOCS3 mRNA	4.09 (2.68 – 6.25)	3.26 (2.21 – 4.56)	2.68 (0.58 – 4.54)	3.17 (2.00 – 4.33)	**0.003**

**Table 5.** Relationship between SP concentrations and T cell-regulating factors. Median for SP quartile, 25^th^ – 75^th^ percentile and p trend value (p*). Significant values after Bonferroni correction are shown in bold font.

	1^st^ quartile (3.50 – 128.4 pg/ml)	2^nd^ quartile (128.4 – 193.0 pg/ml)	3^rd^ quartile (193.0 – 254.7 pg/ml)	4^th^ quartile (254.7 – 1274.0 pg/ml)	p*
IL-4 mRNA	8.56 (4.03 – 25.71)	7.90 (3.96 – 19.9)	7.79 (3.08 – 16.7)	8.62 (4.47 – 23.40)	0.353
IL-4 protein	5.94 (1.50 – 11.46)	5.00 (1.5 – 7.68)	6.30 (1.50 – 9.22)	5.80 (1.50 – 9.48)	0.492
IL-5 protein	2.16 (1.50 – 2.50)	1.50 (1.50 – 2.16)	1.50 (1.50 – 2.34)	1.50 (1.50 – 2.14)	0.097
IL-9 protein	9.60 (2.00 – 20.14)	6.82 (2.00 – 14.28)	9.60 (2.00 – 15.76)	9.60 (2.00 – 16.49)	0.331
IFN-γ protein	5.68 (2.00 – 11.6)	7.32 (2.00 – 11.66)	7.36 (2.00 – 14.79)	10.36 (1.00 – 16.2)	**0.0005**
GATA3 mRNA	6.71 (4.27 – 14.42)	7.57 (3.36 – 13.06)	4.12 (2.93 – 10.86)	4.64 (3.13 – 8.78)	**0.0009**
Tbet mRNA	0.18 (0.11 – 0.40)	0.16 (0.05 – 0.40)	0.19 (0.02 – 0.35)	0.21 (0.08 – 0.33)	0.220
FOXP3 mRNA	0.51 (0.33 – 1.07)	0.56 (0.24 – 0.87)	0.39 (0.21 – 0.85)	0.48 (0.29 – 0.98)	0.176
SOCS1 mRNA	5.55 (3.34 – 10.83)	5.60 (3.85 – 12.34)	5.07 (2.87 – 13.81)	5.17 (3.06 – 14.15)	0.238
SOCS3 mRNA	4.00 (2.69 – 6.14)	3.24 (0.99 – 5.38)	3.08 (1.52 – 5.62)	2.77 (1.88 – 4.08)	**0.001**

**Table 6.** Frequency (% (n)) and risk (adjusted* OR with 95% CI) of allergic sensitization (specific IgE) depending on the SOM concentration. Significant values after Bonferroni correction are shown in bold font.

	SOM				
	1^st^ quartile (3.5 – 21.5 pg/ml)	2^nd^ quartile (21.5 – 30.3 pg/ml)	3^rd^ quartile (30.3 – 42.8 pg/ml)	4^th^ quartile (42.8 – 233.6 pg/ml)	p trend
Food allergens	6.25% (5) 1.00	7.40% (6) 1.45 (0.39 – 5.41)	16.45% (13) 4.71 (1.39 – 15.91)	20.98% (17) 4.93 (1.57 – 15.47)	**0.001**
Hen’s egg	2.50% (2) 1.00	1.23% (1) 0.57 (0.04 – 8.08)	6.23% (5) 4.28 (0.67 – 27.40)	4.39% (4) 2.74 (0.43 – 17.34)	0.191
Cow’s milk	1.25% (1) 1.00	2.46% (2) 2.39 (0.18 – 30.78)	10.12% (8) 11.0 (1.25 – 97.44)	11.11% (9) 8.81 (1.01 – 76.72)	**0.002**
Peanut	1.25% (1) 1.00	1.23% (1) 1.54 (0.07 – 34.21)	3.79% (3) 6.28 (0.54 – 72.01)	8.64% (7) 16 (1.67 – 154.5)	0.008
Inhalable allergens	22.5% (18) 1.00	25.9% (2) 1.30 (0.56 – 3.00)	35.4% (28) 2.48 (1.10 – 5.59)	30.9% (25) 1.99 (0.90 – 4.39)	0.127
Derp1	13.75% (11) 1.00	16.04% (13) 1.27 (0.47 – 3.44)	18.98% (15) 2.02 (0.77 – 5.29)	17.28% (14) 1.48 (0.58 – 3.75)	0.467
Cat	1.25% (1) 1.00	7.40% (6) 7.29 (0.79 – 66.62)	7.59% (6) 6.75 (0.67 – 67.56)	8.64% (7) 8.49 (0.93 – 77.24)	0.064
Dog	1.25% (1) 1.00	4.93% (4) 3.04 (0.29 – 31.50)	3.79% (3) 4.91 (0.41 – 58.35)	6,17% (5) 5.53 (0.59 – 51.29)	0.166
Timothy grass	6.25% (5) 1.00	11.11% (9) 1.73 (0.50 – 6.11)	21.51% (17) 4.58 (1.47 – 14.28)	18.51% (15) 4.13 (1.33 – 12.85)	0.007
Rye	6.25% (5) 1.00	9.87% (8) 1.68 (0.47 – 6.03)	13.92% (11) 3.34 (1.01 – 11.09)	18.51% (15) 4.17 (1.35 – 12.87)	0.012
Birch	5.00% (4) 1.00	11.11% (9) 2.59 (0.71 – 9.46)	15.18% (12) 3.85 (1.07 – 13.82)	12.34% (10) 3.25 (0.90 – 11.69)	0.095
Mugwort	6.25% (5) 1.00	3.70% (3) 0.58 (0.12 – 2.73)	5.06% (4) 1.04 (0.24 – 4.40)	11.11% (9) 2.52 (0.75 – 8.49)	0.192

*adjusted for atopic family history, sex, social state, pet keeping, smoking during pregnancy, breast feeding and month of birth. OR = odds ratio.

**Table 7.** Frequency (% (n)) and risk (adjusted* OR with 95% CI) of allergic sensitization (specific IgE) depending on the VIP concentration. Significant values after Bonferroni correction are shown in bold font.

	VIP				
	1^st^ quartile (3.5 – 141.2 pg/ml)	2^nd^ quartile (141.2 – 196.7 pg/ml)	3^rd^ quartile (197.7 – 249.2 pg/ml)	4^th^ quartile (249.2 – 561.2 pg/ml)	p trend
Food allergens	7.40% (6) 1.00	8.86% (7) 1.34 (0.40 – 4.48)	12.5% (10) 1.48 (0.48 – 4.55)	22.22% (18) 3.50 (1.25 – 9.82)	**0.0038**
Hen’s egg	3.70% (3) 1.00	2.53% (2) 0.62 (0.09 – 4.30)	1.25% (1) 0.15 (0.01 – 2.03)	7.40% (6) 2.17 (0.46 – 10.25)	0.297
Cow’s milk	3.70% (3) 1.00	3.79% (3) 1.19 (0.21 – 6.60)	3.75% (3) 0.88 (0.16 – 4.90)	13.58% (11) 4.00 (1.02 – 15.67)	0.014
Peanut	1.23% (1) 1.00	3.79% (3) 3.11 (0.29 – 32.74)	6.25% (5) 4.67 (0.48 – 45.11)	3.70% (3) 2.94 (0.28 – 31.01)	0.297
Inhalable allergens	30.86% (25) 1.00	20.25% (16) 0.61 (0.27 – 1.35)	30.00% (24) 0.91 (0.42 – 1.97)	33.33% (27) 1.07 (0.52 – 2.21)	0.451
Derp1	18.51% (15) 1.00	13.92% (11) 0.81 (0.32 – 2.05)	16.25% (13) 0.81 (0.33 – 1.98)	17.28% (14) 0.84 (0.36 – 1.96)	0.937
Cat	8.64% (7) 1.00	2.53% (2) 0.37 (0.06 – 2.01)	5.00% (4) 0.75 (0.19 – 2.94)	8.64% (7) 1.09 (0.33 – 3.53)	0.842
Dog	4.93% (4) 1.00	2.53% (2) 0.65 (0.10 – 4.15)	3.75% (3) 1.16 (0.21 – 6.16)	4.93% (4) 0.92 (0.20 – 4.03)	0.903
Timothy grass	12.34% (10) 1.00	8.86% (7) 0.82 (0.28 – 2.42)	13.75% (11) 1.41 (0.52 – 3.83)	22.22% (18) 2.32 (0.92 – 5.85)	0.048
Rye	7.40% (6) 1.00	7.59% (6) 1.12 (0.33 – 3.81)	13.75% (11) 2.23 (0.73 – 6.85)	19.75% (16) 3.22 (1.12 – 9.29)	0.008
Birch	7.40% (6) 1.00	8.86 % (7) 1.14 (0.35 – 3.73)	13.75 % (11) 1.62 (0.53 – 4.95)	13.58 % (11) 1.95 (0.63 – 5.98)	0.132
Mugwort	6.17 % (5) 1.00	7.59% (6) 1.38 (0.38 – 4.96)	3.75% (3) 0.57 (0.12 – 2.72)	8.64% (7) 1.44 (0.41 – 5.05)	0.768

*adjusted for atopic family history, sex, social state, pet keeping, smoking during pregnancy, breast feeding and month of birth. OR = odds ratio.
